# Comparison of Morbidity and Mortality Outcomes between Hybrid Palliation and Norwood Palliation Procedures for Hypoplastic Left Heart Syndrome: Meta-Analysis and Systematic Review

**DOI:** 10.3390/jcm13144244

**Published:** 2024-07-20

**Authors:** Christopher Iskander, Ugonna Nwankwo, Krithika K. Kumanan, Saurabh Chiwane, Vernat Exil, Lia Lowrie, Corinne Tan, Charles Huddleston, Hemant S. Agarwal

**Affiliations:** 1 Division of Pediatric Cardiology, Cardinal Glennon Children’s Hospital, Saint Louis, MO 63104, USA; christopher.iskander@health.slu.edu (C.I.); ugonna.nwankwo@slucare.ssmhealth.com (U.N.); vernat.exil@slucare.ssmhealth.com (V.E.); 2 Advanced Data Health Institution, Saint Louis University, Saint Louis, MO 63104, USA; krithikakumanan@gmail.com; 3 Division of Pediatric Critical Care Medicine, Loma Linda University, Loma Linda, CA 92354, USA; schiwane@llu.edu; 4 Division of Pediatric Critical Care Medicine, Cardinal Glennon Children’s Hospital, Saint Louis, MO 63104, USA; lia.lowrie@health.slu.edu; 5 Department of Pediatric Cardio-Thoracic Surgery, Cardinal Glennon Children’s Hospital, Saint Louis, MO 63104, USA; corrine.tan@health.slu.edu (C.T.); charles.huddleston@slucare.ssmhealth.com (C.H.)

**Keywords:** meta-analysis, hypoplastic left heart syndrome, Norwood operation, sano, BT shunt, hybrid palliation/hybrid procedure, bilateral pulmonary artery banding, outcomes, death/mortality, morbidity

## Abstract

**Background/Objectives**: Hybrid palliation (HP) procedures for hypoplastic left heart syndrome (HLHS) are increasing. Our objective was to compare mortality and morbidity following HP and NP (Norwood palliation) procedures. **Methods**: Systematic review and meta-analysis of HLHS patients of peer-reviewed literature between 2000 and 2023. Mortality and/or heart transplantation in HP versus NP in the neonatal period, interstage period, and at 1, 3 and 5 years of age, and morbidity including completion of Stage II and Stage III palliation, unexpected interventions, pulmonary artery pressures, right ventricle function, neurodevelopmental outcomes and length of hospital stay were evaluated. **Results**: Twenty-one (meta-analysis: 16; qualitative synthesis: 5) studies evaluating 1182 HLHS patients included. HP patients had higher interstage mortality (RR = 1.61; 95% CI: 1.10–2.33; *p* = 0.01) and 1-year mortality (RR = 1.22; 95% CI: 1.03–1.43; *p* = 0.02) compared to NP patients without differences in 3- and 5-years mortality. HP procedure in high-risk HLHS patients had lower mortality (RR = 0.48; 95% CI: 0.27–0.87; *p* = 0.01) only in the neonatal period. HP patients underwent fewer Stage II (RR = 0.90; 95% CI: 0.81–1.00; *p* = 0.05) and Stage III palliation (RR = 0.78; 95% CI: 0.69–0.90; *p* < 0.01), had more unplanned interventions (RR = 3.38; 95% CI: 2.04–5.59; *p* < 0.01), and longer hospital stay after Stage I palliation (weighted mean difference = 12.88; 95% CI: 1.15–24.62; *p* = 0.03) compared to NP patients. **Conclusions**: Our study reveals that HP, compared to NP for HLHS, is associated with increased morbidity risk without an improved survival rate.

## 1. Introduction

Congenital heart defects (CHD) account for approximately 20% of all major structural birth defects and hypoplastic left heart syndrome (HLHS) comprises 8–12% of CHD [1]. HLHS is characterized by an underdevelopment of the left-sided cardiac structures, compromised systemic cardiac output and a single right ventricle [2]. It was first described by Lev in 1952 and given the name “hypoplastic left heart syndrome” by Noonan and Nadas in 1958 [3,4].

HLHS is the most severe form of CHD, associated with significant morbidity and mortality. It is universally fatal and 95% of untreated patients with HLHS die early [5]. Treatment options for HLHS have continued to evolve and include comfort care, cardiac transplantation, Norwood palliation (NP) or hybrid palliation (HP) [6]. The role of comfort care as a treatment modality for HLHS is on the decline [7]. It remains a choice for HLHS patients with very low birth weight, prematurity or the presence of a chromosomal anomaly [8]. Death occurs in 92% of these infants while hospitalized and in 8% while in hospice [8]. Cardiac transplantation is another treatment modality for neonatal HLHS patients with the possibility of an ABO-incompatible transplantation [9]. Outcomes following cardiac transplantation in neonatal HLHS patients are excellent when they receive it in early life and avoid clinical decline or mortality while awaiting transplantation [10]. Cardiac transplantation in the neonatal period remains limited due to the scarcity of a donor pool [11].

The introduction of NP in the 1980s has made the three-stage palliative surgical approach the most common treatment option for HLHS management [12,13]. It is the preferred operative choice in 80–90% of HLHS neonates in the USA and Europe [14,15,16]. NP or Stage 1 palliation takes place in the first few days of life with the pulmonary blood flow established either by a modified Blalock–Taussig shunt (BT) or right ventricle to pulmonary artery shunt (Sano shunt). The Glenn procedure, or the second stage (Stage II), is performed at 4–6 months of age, and the Fontan operation, or the third and final stage (Stage III), is performed around 18–24 months of age. Advancements in perioperative care of HLHS have improved the survival rate in HLHS [17]. HLHS outcomes in the neonatal period, however, continue to remain poor compared to those in patients with less severe CHD [18]. Mortality following NP remains at 15–17% in high-volume centers and 20–40% in combined pediatric heart programs [19,20,21,22,23]. The longer-term survival rates after Stage III palliation have also plateaued at 54–60% [24,25,26]. The lack of a reduction in mortality following NP has been attributed to numerous high-risk factors, including low birth weight, prematurity, genetic abnormalities, cardiac conditions like intact atrial septum, severe atrioventricular valve insufficiency or noncardiac conditions [27,28]. There has been an increasing concern about the impact of the NP and its consequences on late neurodevelopmental outcomes in surviving patients [17,29].

A lack of further reduction in HLHS mortality following NP initiated a search for newer surgical techniques to treat HLHS. HP was initially described in the 1990s by Gibbs et al. and subsequently modified by Akintuerk et al. as an alternative palliative approach for HLHS [30,31]. There has been an increasing trend toward HP procedures in the past decade, with approximately 13–20% of pediatric heart centers in the USA and Europe and >50% of heart programs in Japan performing it [8,14,15,32,33]. It is the primary treatment modality for all HLHS patients at some centers, while in other centers, it is being offered to high-risk HLHS patients [5,34,35]. The technique of HP has evolved, including placement of bilateral pulmonary artery (PA) bands, stenting of the ductus arteriosus (or prostaglandin infusion), and/or atrial septostomy in the neonatal period, followed by arch reconstruction and a bidirectional cavo-pulmonary shunt as Stage II palliation at 4–6 months and Fontan operation at 18–24 months of age. Avoidance of cardiopulmonary bypass (CPB) and deep hypothermic circulatory arrest (DHCA) or antegrade cerebral perfusion (ACP) during the HP have been proposed to be beneficial for HLHS outcomes.

Studies from pediatric cardiac programs that perform NP and HP for HLHS patients are mostly retrospective and observational in nature, reporting outcomes in a small number of patients [36,37,38,39,40,41,42,43,44,45,46,47,48,49,50,51,52,53,54,55,56]. Large databases and multicentric studies have revealed mixed results for short-term and long-term outcomes for NP versus HP in the neonatal period [14,15,16,57]. A comparison of these results has limitations such as institutional differences, dissimilar methods of classifying patient risk factors, different learning and team experiences and additional medical or surgical problems. We thus undertook a systematic review and meta-analysis of studies that have reported both NP and HP procedures in the HLHS patient population to compare morbidity and mortality. We assessed mortality and/or transplantation following NP or HP in the neonatal and interstage periods and at 1 year, 3 years and 5 years of age. We also assessed mortality and/or transplantation in high-risk HLHS patients for these palliative procedures in the same period. We studied morbidity, including successful completion of Stage II and Stage III palliation, number of unplanned interventions, pulmonary artery pressures and right ventricular function at Stage II and Stage III palliation, neurodevelopmental outcomes and utilization of hospital resources, including length of intensive care unit (ICU) and hospital stay, for HLHS patients who underwent NP or HP.

## 2. Materials and Methods

### 2.1. Search Strategy

The systematic review and meta-analysis were conducted according to the Preferred Reporting Items for Systematic Reviews and Meta-Analyses (PRISMA) guidelines [58]. A comprehensive literature search was carried out with the assistance of an information specialist at the Saint Louis University Medical Library (Saint Louis, MO, USA). A Medical Subject Headings (MeSH) search for hypoplastic left heart syndrome, Norwood, Sano, or Blalock shunt, or hybrid palliation or pulmonary artery banding was performed in the publication databases PubMed, ProQuest, Google Scholar, and EBSCO (CINAHL, CINAHL Plus, Medline) for citations between 2000 and 2023. A manual search of reference lists of each included article was carried out for additional studies.

### 2.2. Search Selection

We used the PICO (Patient/Population, Intervention, Comparison and Outcomes) framework to establish the inclusion criteria for all relevant original articles: (1) Population: patients with HLHS; (2) Intervention: HP; (3) Comparison: NP: Sano shunt or BT shunt; (4) Outcome: primary outcome of death and/or heart transplantation. Case reports, opinion articles, experimental studies, and case series with less than 10 patients were excluded. Conference abstracts, studies with mixed/non-HLHS lesions, review articles, guidelines, non-English language papers and animal studies were also excluded. The following steps were taken for study selection: (1) identification of titles of studies through database searches; (2) exclusion of duplicates; (3) abstracts screening and selection; (4) assessment for eligibility through full-text articles; and (5) final inclusion in the study. Initially, titles and abstracts of the retrieved studies were screened independently by three reviewers (C.I., N.U. and H.S.A.). The full text of all the potentially eligible studies was retrieved and independently assessed for eligibility by the same three investigators. Any disagreement between the investigators over the eligibility of a specific study was resolved through consensus and discussion. For multiple studies with overlapping cohorts, each study was critically appraised, and only the one with the largest sample size and complete available data was used for analysis.

### 2.3. Data Extraction

All included studies were reviewed for study design, number of patients undergoing HP or NP, demographics, a primary outcome of either death or heart transplant. The primary outcome was assessed at hospital discharge following HP or NP, interstage period, at 1 year, 3 years and 5 years of age. Death and/or heart transplantation were also assessed for high-risk patients for similar periods. Low birth weight (<2.5 kg), prematurity, genetic abnormalities, cardiac conditions like intact atrial septum, severe atrioventricular valve insufficiency or noncardiac conditions were included as high-risk factors [27,28]. Secondary outcomes reviewed were number of patients undergoing Stage II (bidirectional Glenn or comprehensive Stage II) palliation and Stage III (Fontan) palliation, number of unplanned interventions, pulmonary artery pressures at Stage II and Stage III palliation, right ventricular function at Stage II and Stage III palliation, intensive care unit (ICU) and hospital length of stay after Stage I and Stage II palliation and neurodevelopmental outcomes at 1 year, 3 years and 5 years of age.

### 2.4. Risk of Bias and Quality Assessment

The Risk of Bias in Non-Randomized Studies of Interventions (ROBINS-1) tool was systematically used to assess the included studies for risk of bias [59]. The studies and their characteristics were classified into low, moderate, serious and critical risk of bias.

### 2.5. Statistical Analysis

The meta-analysis was conducted with R Statistical Software (version 4.2.1, Foundation for Statistical Computing) using the random-effects model. The risk ratio and its 95% confidence interval (CI) were used to measure the risk of events between the two palliative procedures and were depicted using forest plots. Weighted mean difference and its 95% CI was used when the results were continuous. Hazard ratios were calculated from time to event data. In articles where the hazard ratio was not reported, it was derived from the Kaplan–Meier curves [60]. A meta-analysis was conducted if at least 3 studies met the criteria for assessing the relationship between the 2 different procedures and the mortality and/or morbidity outcomes. An I^2^ statistic was generated to determine the inconsistencies in effect size across studies and describe heterogeneity [61]. For the purposes of this meta-analysis, we considered I^2^ < 25% as having a consistent effect on the outcome variable and low heterogeneity, >50% as highly varied effects and with high heterogeneity and 25% to 50% as moderate heterogeneity. The random effects model was then applied to combine the estimated effects with the assumption that actual effect sizes varied among studies. Funnel plots were generated and statistically assessed by the Begg test and the Egger test for publication bias [62,63].

## 3. Results

### 3.1. Study Selection and Characteristics

**Figure 1:**
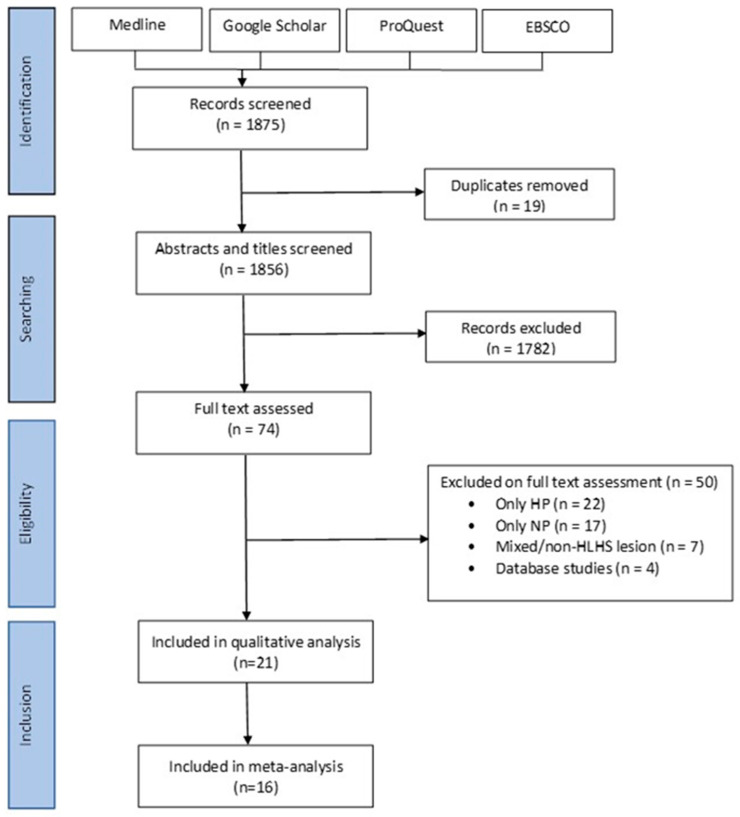
Flow diagram of search strategy of studies conducted according to the Preferred Reporting Items for Systematic Reviews and Meta-Analyses (PRISMA) guidelines [58]. HP: Hybrid Palliation, NP: Norwood Palliation, HLHS: Hypoplastic Left Heart Syndrome.

A total of 1856 studies were screened based on their titles and abstracts. Full-text screening was performed on 74 articles. Nine additional articles were found on the iteration of references. Four studies were eliminated for overlapping cohorts, and data from one study was merged with another study from the same center [52,53,54,55,56]. Twenty-one studies met the inclusion criteria for meta-analysis and systematic review (Figure 1). Of these, 16 studies were included for meta-analysis, and five were included for qualitative analysis [36,37,38,39,40,41,42,43,44,45,46,47,48,49,50,51,64,65,66,67,68] (Table 1). All included studies were conducted in America, Europe, and Japan (Table 1).

### 3.2. Risk of Bias and Quality Assessment

**Figure 2:**
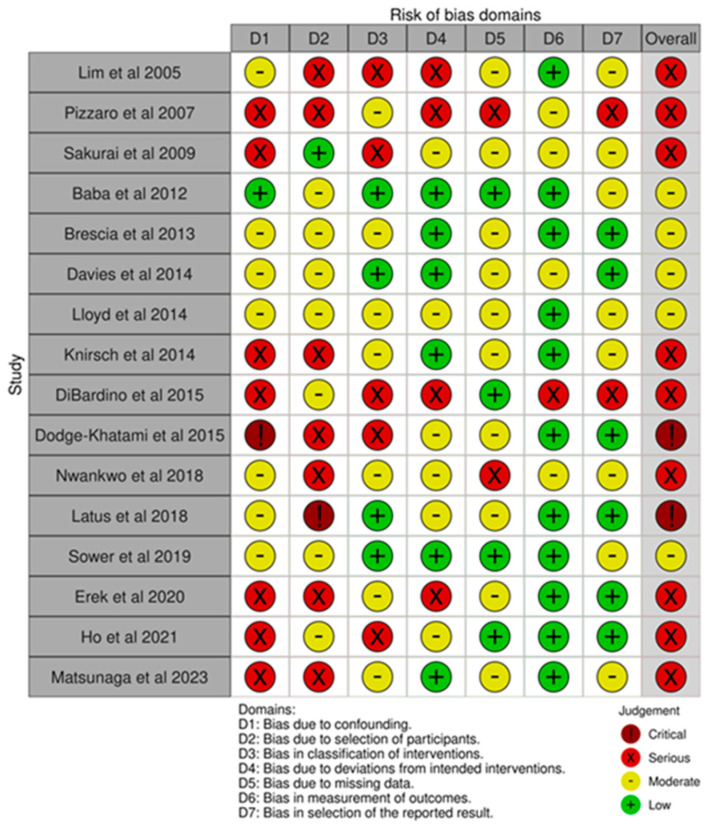
Risk of bias of studies included in meta-analysis as per The Risk of Bias in Non-Randomized Studies of Interventions (ROBINS-1) tool [36,37,38,39,40,41,42,43,44,45,46,47,48,49,50,51,59].

The studies included in our meta-analysis and systematic review were retrospective, observational studies (Table 1). The risk of bias was moderate in five, serious in nine and critical in two studies per the ROBINS-1 tool (Figure 2). Publication bias assessed using a funnel plot and Egger’s test disclosed no asymmetry around the axis for all measured proportions (Figures S1–S3).

### 3.3. Baseline Patient Demographics

**Figure 3:**
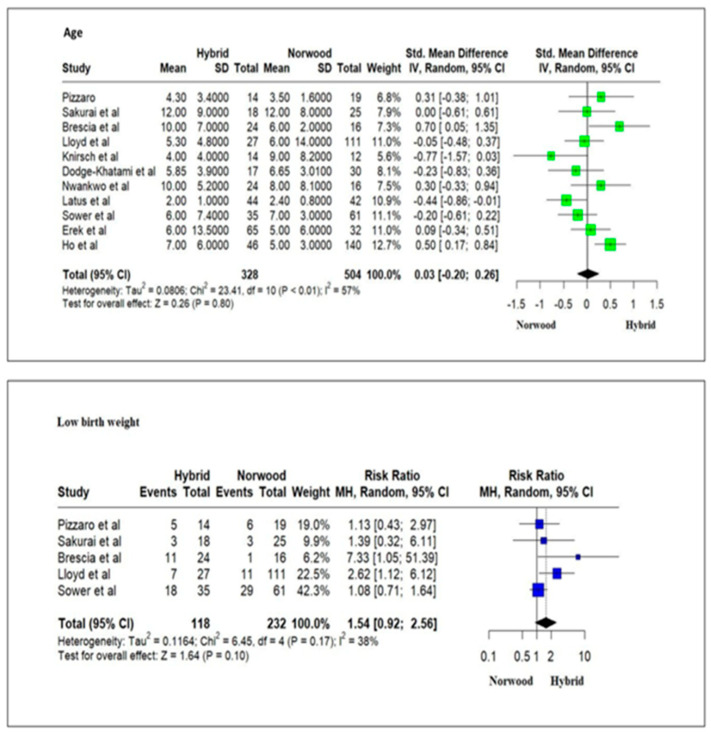
Forest plot of day of life for the operation and presence of low birth weight (<2.5 kg) for HLHS patients undergoing hybrid or Norwood palliation [37,38,40,42,43,45,46,47,48,49,50,56].

The studies included 1182 HLHS patients. Of these, 479 (41%) patients underwent HP, and 703 (59%) underwent NP in the neonatal period. There was no significant difference in the day of life when these patients were operated on or the presence of low birth weight (<2.5 kg) between the HP and NP cohorts (Figure 3).

### 3.4. Primary Outcomes: Mortality

**Figure 4:**
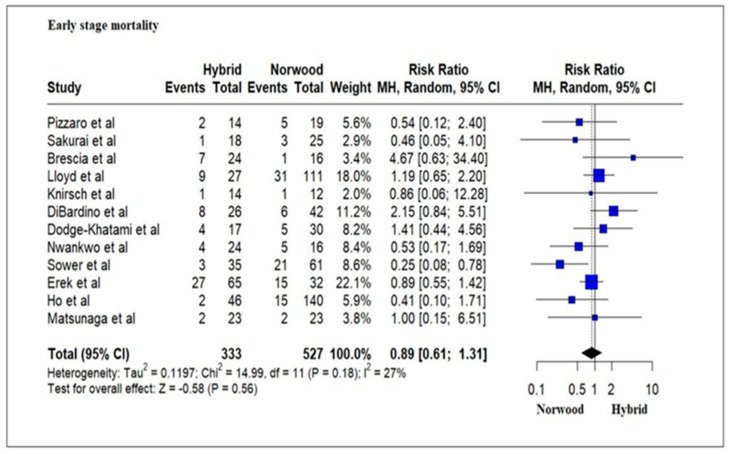
Forest plot of in-hospital mortality and/or transplantation in HLHS patients undergoing hybrid or Norwood palliation in the neonatal period [37,38,40,42,43,45,46,47,49,50,51,56].

**Figure 5:**
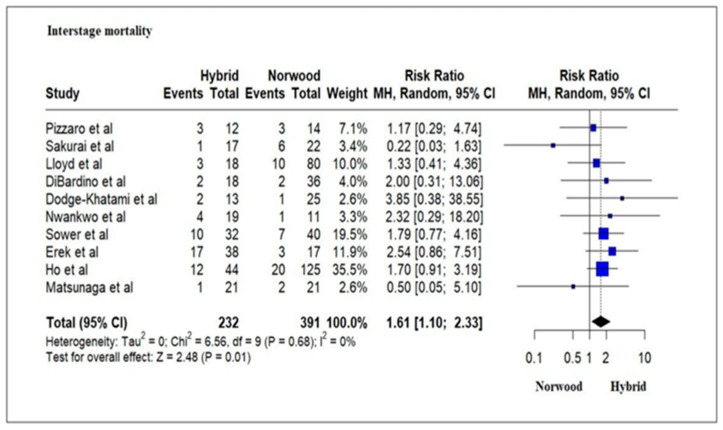
Forest plot of interstage mortality and/or transplantation in HLHS patients undergoing hybrid or Norwood palliation in the neonatal period [37,38,42,44,45,46,48,49,50,51].

**Figure 6:**
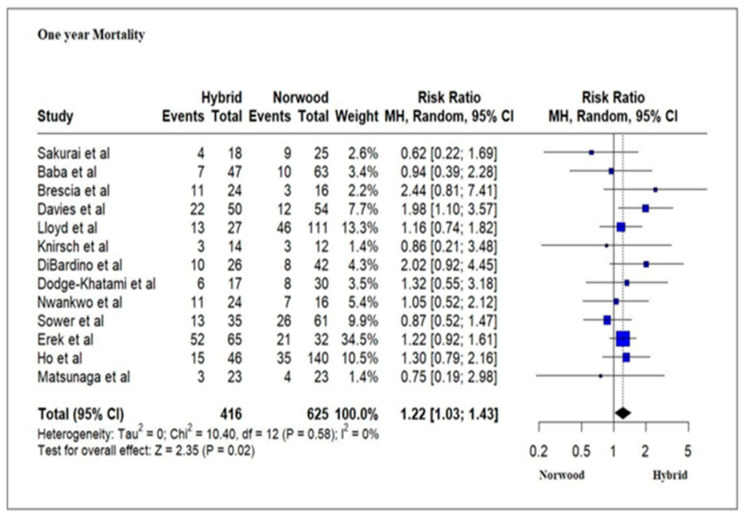
Forest plot of 1-year mortality and/or transplantation in HLHS patients undergoing hybrid or Norwood palliation in the neonatal period [38,39,40,41,42,43,44,45,46,48,49,50,51,52,53,54,55,56].

**Figure 7:**
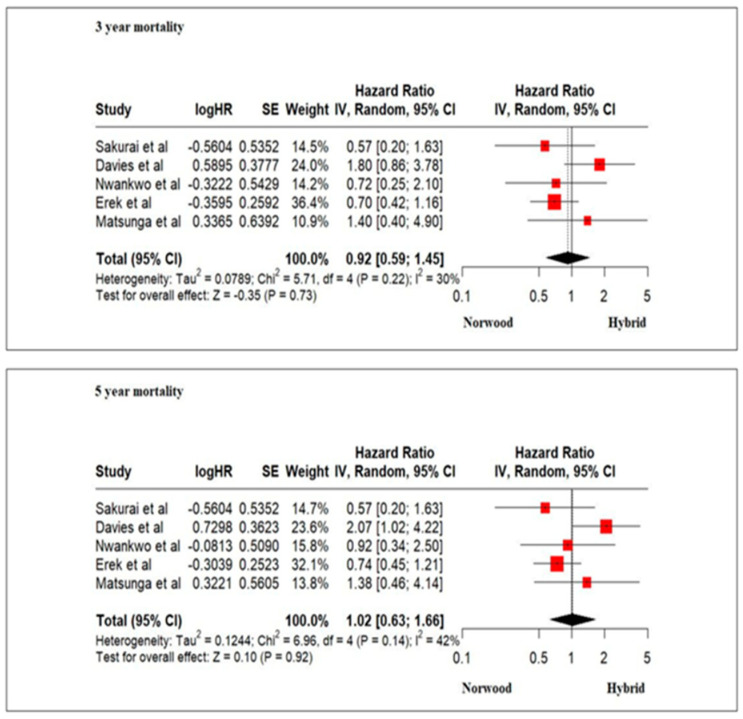
Forest plot of 3-year and 5-year mortality and/or transplantation in HLHS patients undergoing hybrid or Norwood palliation in the neonatal period [38,41,46,49,51,52].

Twelve studies evaluating 860 HLHS patients reported an in-hospital mortality and transplantation rate of 21% (70/333) for the HP cohort and 20.8% (110/527) for the NP cohort without any significant difference (RR = 0.88; 95% CI: 0.61–1.28, *p* = 0.56) (Figure 4). Ten studies evaluating 623 HLHS patients reported an interstage mortality and transplantation rate ofs 23.7% (55/232) for the HP cohort and 14.06% (55/391) for the NP cohort, which was statistically significant (RR = 1.61; 95% CI:1.10–2.33; *p* = 0.01) (Figure 5). Thirteen studies evaluating 1041 HLHS patients reported a one-year mortality and transplantation rate of 43.99% for the HP cohort and 30.72% (192/625) for the NP cohort, which was statistically significant (RR = 1.22; 95% CI: 1.03–1.43; *p* = 0.02) (Figure 6). Five studies evaluating 330 HLHS patients (HP cohort *n* = 180 and NP cohort *n* = 150) reported mortality and need for transplantation at 3 years and 5 years of age. There was no significant difference for mortality and/or transplantation at 3 years (Hazard Ratio (HR) = 0.92; 95% CI: 0.59–1.45; *p* = 0.73) and 5 years of age (HR = 1.02; 95% CI: 0.63–1.66; *p* = 0. 92) among the HP cohort and the NP cohort patients (Figure 7).

**Figure 8:**
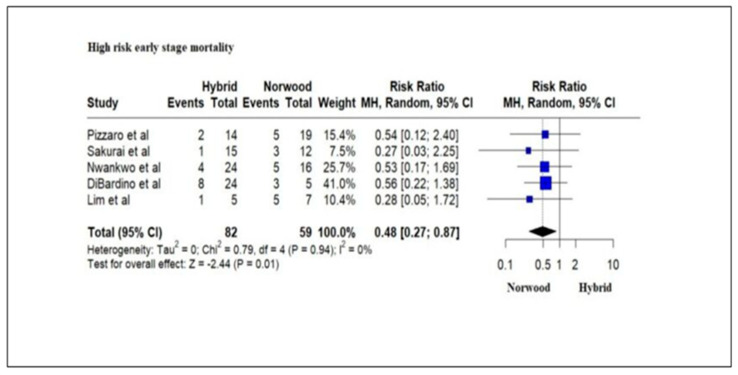
Forest plot of in-hospital mortality and/or transplantation in high-risk HLHS patients undergoing hybrid or Norwood palliation in the neonatal period [36,37,38,44,46].

**Figure 9:**
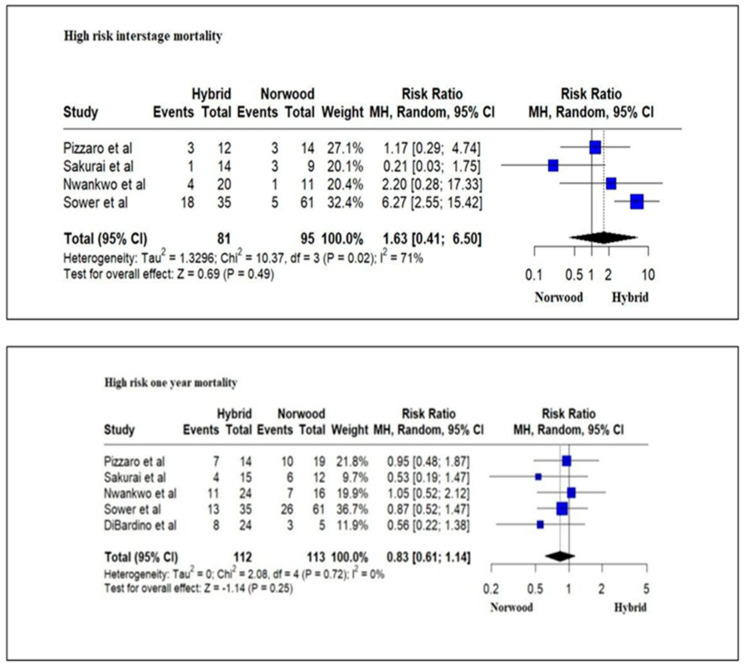
Forest plot of inter-stage and 1-year mortality and/or transplantation in high-risk HLHS patients undergoing hybrid or Norwood palliation in the neonatal period [37,38,44,46,48].

Five studies evaluating 141 high-risk HLHS patients (low birth weight (<2.5 kg), prematurity < 37 weeks of gestation, genetic abnormalities, cardiac conditions such as an intact atrial septum or severe valve insufficiency, noncardiac conditions) reported an in-hospital mortality and/or transplantation rate of 19.5% (16/82) for the HP cohort and 35.59% (21/59) for the NP cohort. The in-hospital mortality and/or transplantation rate was significantly lower for the HP cohort as compared to the NP cohort (RR = 0.48; 95% CI: 0.27–0.87; *p* = 0.01) (Figure 8). However, there was no significant difference in the mortality and/or transplantation rate during the interstage period (RR = 1.63; 95% CI: 0.41–6.50; *p* = 0.49) and at 1 year of age between the HP cohort and the NP cohort (RR = 0.83; 95% CI: 0.61–1.14; *p*-value = 0.25) (Figure 9).

### 3.5. Secondary Outcomes: Morbidity

**Figure 10:**
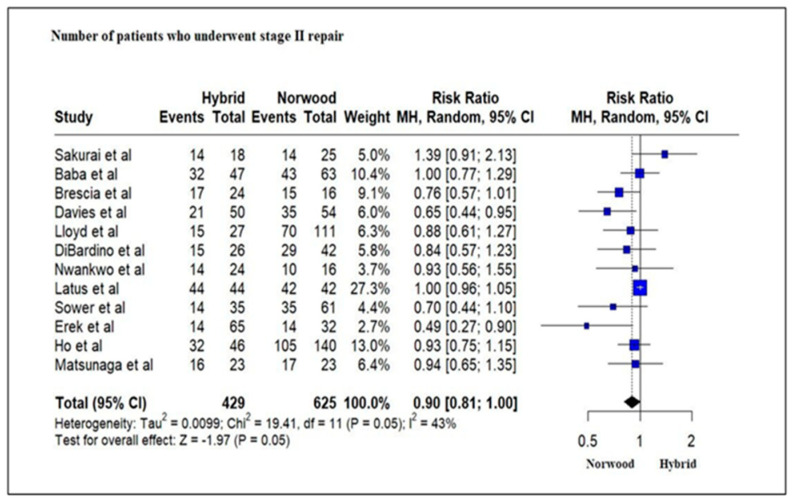
Forest plot of the number of patients undergoing Stage II palliation in HLHS patients undergoing hybrid or Norwood palliation in the neonatal period [38,39,40,41,42,44,46,47,48,49,51,52,53,54,55].

**Figure 11:**
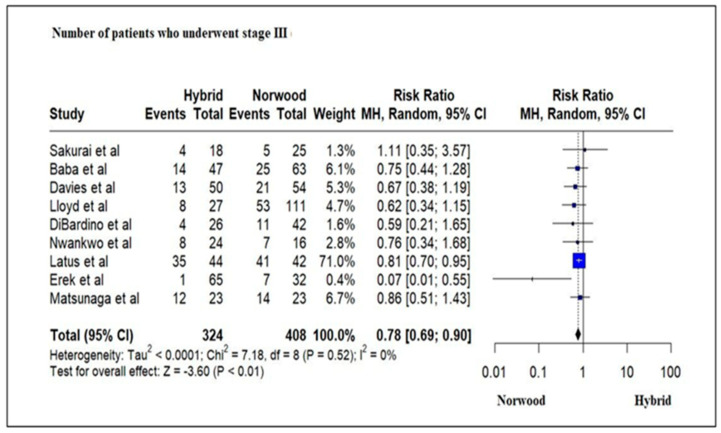
Forest plot of the number of patients undergoing Stage III palliation in HLHS patients undergoing hybrid or Norwood palliation in the neonatal period [38,39,41,42,44,46,47,49,51,52,53,54,55].

Twelve studies evaluating 1054 HLHS patients revealed that a greater number of patients in the NP cohort (429/625, 68%) underwent Stage II palliation as compared to the HP cohort (248/429, 58%), although this was not statistically significant (RR = 0.90; 95% CI: 0.81–1.00; *p* = 0.05) (Figure 10). Nine studies evaluating 732 HLHS patients revealed that 31% of patients in the HP cohort underwent Stage III (Fontan procedure) palliation as compared to 45% in the NP cohort, which was statistically significant (RR = 0.78; 95% CI: 0.69–0.90; *p* < 0.01) (Figure 11).

**Figure 12:**
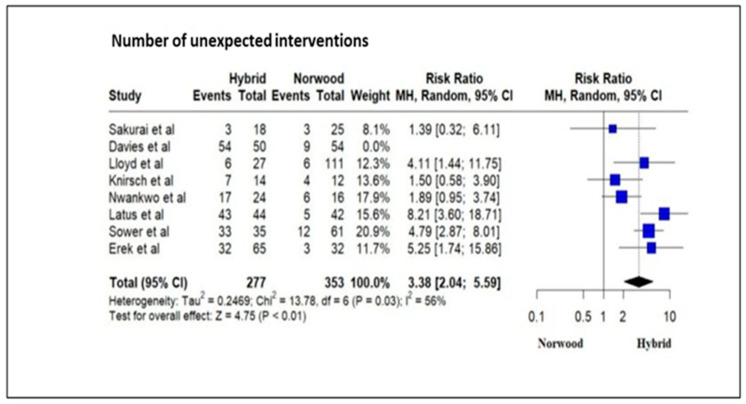
Forest plot of the number of unexpected interventions in HLHS patients undergoing hybrid or Norwood palliation in the neonatal period [38,41,42,43,46,47,48,49,52,56].

Eight studies evaluating 632 HLHS patients revealed a significantly increased number of unexpected interventions (cardiac catheter intervention and surgical) following Stage 1 palliation in the HP cohort as compared to the NP cohort (RR = 3.38; 95% CI: 2.04–5.59; *p* < 0.01) (Figure 12).

**Figure 13:**
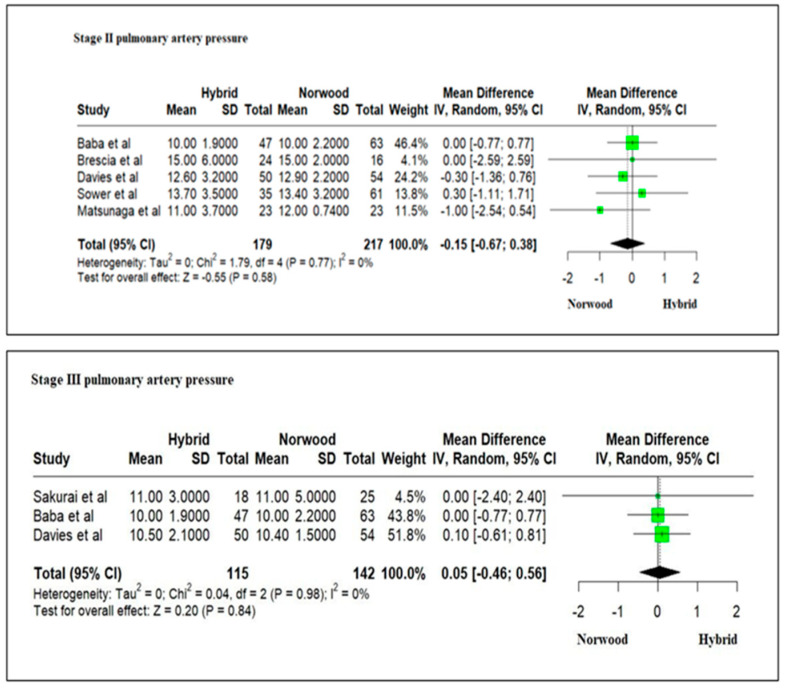
Forest plot of mean pulmonary artery pressures at Stage II and Stage III palliation in HLHS patients undergoing hybrid or Norwood palliation in the neonatal period [38,39,40,41,48,51,52,53,54,55].

Five studies evaluating pulmonary artery pressures in 396 HLHS patients at Stage II palliation revealed mean pulmonary artery pressures (mPAP): 12.46 ± 3.66 mm Hg in the HP cohort as compared to 12.66 ± 2.06 mm Hg in the NP cohort without any significant difference (RR = −0.15; 95% CI: −0.67–0.38; *p* = 0.58) (Figure 13). Three studies evaluating pulmonary artery pressures in 257 HLHS patients at Stage III palliation revealed mPAP: 10.5 ± 2.33 mm Hg in the HP cohort as compared to 10.46 ± 2.9 mm Hg in the NP cohort without any significant difference (RR = 0.05; 95% CI: −0.46–0.56; *p* = 0.84) (Figure 13).

**Figure 14:**
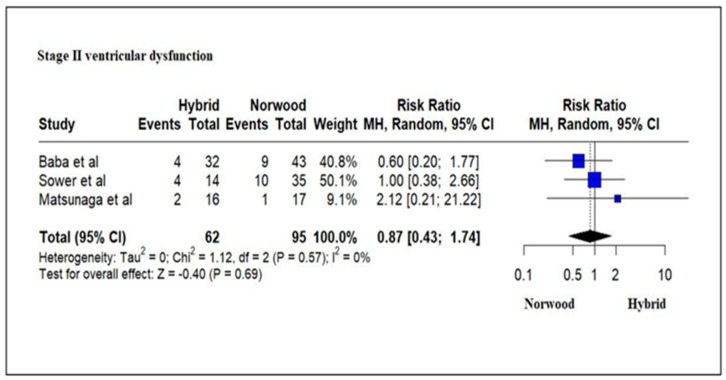
Forest plot of ventricular dysfunction in HLHS patients undergoing hybrid or Norwood palliation in the neonatal period [39,48,51,53,54,55].

Three studies evaluating 157 HLHS patients revealed ventricular dysfunction (mild/moderate/severe) by echocardiography in 16% (10/62) of patients in the HP cohort as compared to 21% (20/95) of patients in the NP cohort at Stage II palliation. There was no significant difference between the two cohorts (RR = 0.87; 95% CI: 0.43–1.74; *p* = 0.69) (Figure 14). A meta-analysis for the presence of ventricular dysfunction at Stage III palliation could not be undertaken due to the lack of enough studies.

**Figure 15:**
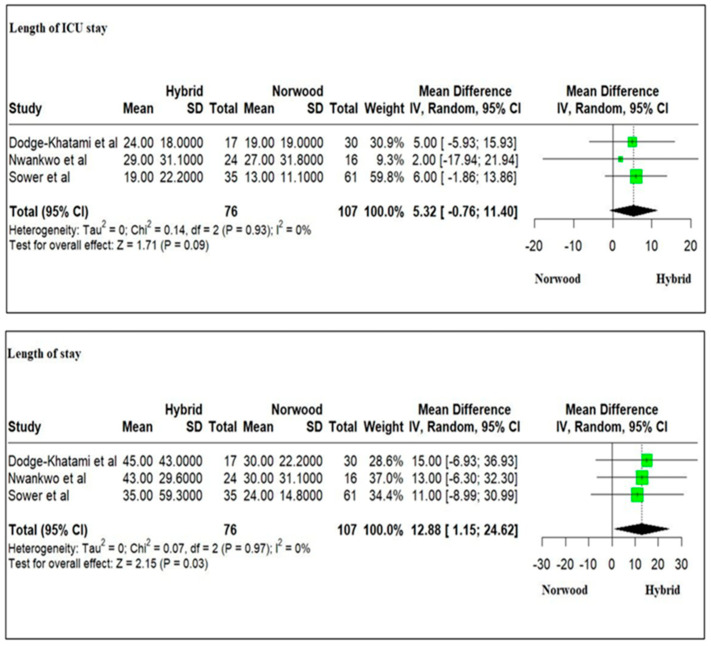
Forest plot of the length of ICU (Intensive Care Unit) and hospital stay at Stage I palliation in HLHS patients undergoing hybrid or Norwood palliation in the neonatal period [45,46,48].

Three studies evaluating 183 HLHS patients revealed a longer ICU stay for the HP cohort as compared to the NP cohort (mean: 24 ± 23.76 days versus 19.6 ± 20.63 days, respectively) and statistically significant longer hospital stay for the HP cohort as compared to the NP cohort following Stage I palliation (weighted mean difference = 12.88; 95% CI: 1.15–24.62; *p* = 0.03) (Figure 15). A meta-analysis for the length of ICU stay, and hospital stay following Stage II palliation could not be undertaken due to the lack of enough studies.

A meta-analysis of neurodevelopmental outcomes at 1 year, 3 years and 5 years for the HP and NP cohort patients could not be undertaken due to the lack of enough studies.

### 3.6. Qualitative Analysis

#### 3.6.1. Ventricular Dysfunction at Stage III Palliation

There was no significant difference in the number of patients having ventricular dysfunction by echocardiography at Stage III palliation in two studies [39,51]. A single-center study of 39 HLHS patients between 2004 and 2010 evaluating ventricular function by echocardiography at Stage III (Fontan) palliation revealed that 4% (1/25) of patients in the NP cohort had mild ventricular dysfunction as compared to 7% (1/14) of patients in the HP cohort [39]. Another study evaluating 31 HLHS patients at Stage III palliation at three centers between 2004 and 2022 revealed that 6% (1/17) of patients in the NP cohort had ventricular dysfunction by echocardiography as compared to 14% (2/14) in the HP cohort [51].

#### 3.6.2. Length of ICU and Hospital Stays after Stage II Palliation

A significantly shorter hospital stay was reported at Stage II palliation for patients undergoing NP as compared to HP [39,40,56]. A single-center study of 22 HLHS patients undergoing Stage II palliation between 2004 and 2008 revealed a similar duration of ICU stay (NP (*n* = 12): mean of 5 days versus HP (*n* = 10): 5 days; *p* = 0.45) and a shorter hospital stay (NP: mean of 14 days versus HP: 30 days; *p* = 0.06) [56]. Another single-center study of 75 HLHS patients undergoing Stage II palliation between 2004 and 2010 revealed a similar length of ICU stay (NP (*n* = 43): mean of 5 days versus HP (*n* = 32): 7 days; *p* = 0.145) and a significantly shorter hospital stay (NP: mean of 9 days versus HP: 16 days; *p* = 0.041) [40]. Lastly, a single-center study of 32 HLHS patients undergoing Stage II palliation revealed a significantly shorter ICU stay [NP (*n* = 15): mean of 5 days versus HP (*n* = 17): 16 days; *p* = 0.001] and hospital length of stay (NP: mean of 8 days versus HP: 24 days; *p* = 0.01) [40].

### 3.7. Neurodevelopmental Outcomes

Although there were limited studies evaluating neurodevelopmental outcomes in HLHS patients undergoing HP versus NP in the neonatal period, they revealed no significant difference at 1 year, 2 years and 4 years between the two palliative procedures [64,66,68]. Assessment of 20 HLHS (NP (*n* = 11), HP (*n* = 9) patients) between 2004 and 2008 at 1 year of age using the Bayley Scales of Infant Development II revealed a significantly lower psychomotor development index (PDI) and mental development index (MDI) outcome in these patients compared to the norm (median PDI 57 (49–99), *p* < 0.001; median MDI 91 (65–109), *p* = 0.002). These indexes were, however, independent of the type of surgical palliative procedure undertaken in the neonatal period (PDI: NP 56.5 (49–81) vs. HP 65 (50–99), *p* = 0.18; MDI: NP 93 (65–109) vs. HP 88 (71–102), *p* = 1.0) [64]. The neurodevelopmental assessment of 44 single ventricle physiology patients at 2 years of age undergoing HP and NP between 2010 and 2015 with the Bayley Scales of Infant and Toddler Development, Third Version (Bayley-III) revealed median cognitive, language, and motor composite scores of 100 (range 65–120), 97 (68–124), and 97 (55–124), respectively [66]. The language composite score was significantly below the norm (*p* = 0.025). There was no significant difference in the Bayley-III scores between NP and HP patients (cognitive score, *p* = 0.91; language score, *p* = 0.58; motor score, *p* = 0.99) [67]. A neurodevelopmental assessment of 16 HLHS patients at 4 years of age undergoing HP and NP between 2004 and 2008 by the Wechsler Primary Preschool Intelligence Scale–III and the Movement-ABC revealed significantly lower cognitive and motor performance than the norm (median IQ: 89 (76–116), *p* = 0.02; total motor score: *p* = 0.002), and was not related to the type of surgical intervention in the neonatal period (IQ: NP 92 (80–104) vs. HP 88 (76–116), *p* = 1.0; motor outcome: *p* > 0.8) [64]. Neuroimaging, including cerebral magnetic resonance, at 2 years of age for 29 HLHS patients undergoing HP and NP between 2012 and 2015 revealed that the total brain volumes in patients with HLHS were significantly smaller compared to controls (HLHS: 893 ± 76 mL vs. control: 1015 ± 148 mL, *p* = 0.005). All three brain compartments (total gray matter, deep gray matter and white matter) were significantly smaller in patients who underwent NP, whereas patients after HP had total and deep gray volumes comparable to controls. Deep grey matter reduction was more pronounced (NP: 38.4 ± 4.1 mL vs. HP: 44.4 ± 3.9 mL, *p* = 0.005) than white matter reduction (NP: 255 ± 19 mL vs. HP: 285 ± 31 mL, *p* = 0.032) among patients undergoing NP versus HP [68].

## 4. Discussion

Our meta-analysis and systematic review revealed that HLHS patients undergoing HP did not have any improved survival benefit as compared to patients undergoing NP at 1, 3 and 5 years of age. HLHS patients with high-risk factors undergoing HP also did not have an improved survival rate at 1 year of age. HLHS patients undergoing HP have an increased risk of morbidity as compared to HP, with a smaller number of patients in the HP cohort undergoing Stage II and Stage III palliation, increased number of unexpected interventions and increased utilization of hospital resources, including prolonged length of hospital stay following Stage I and Stage II palliation. Likewise, there was a lack of improved neurodevelopmental outcomes for the HP cohort patients as compared to NP cohort patients at 1, 2 and 4 years of age.

Our meta-analysis revealed no significant difference in in-hospital mortality following the Norwood palliative procedure versus the hybrid procedure in the neonatal period. Previous studies of HLHS patients undergoing HP and NP from The Society of Thoracic Surgeons Congenital Heart Surgery Database (STS CHSD) between 2010 to 2012 and between 2012 and 2017, the European Congenital Heart Surgeons Association (ECHSA) Congenital Database between 2013 and 2017 and the Pediatric Health Information System database between 1998 and 2012 have reported variable results for in-hospital mortality [13,15,57]. Numerous factors, including patient selection, risk factors, institutional differences, initial learning curve, database limitations and additional medical or surgical issues, may be responsible for these variable results [69].

Our meta-analysis results revealed a significantly increased interstage mortality rate for the HP cohort as compared to the NP cohort. Previous studies of patients undergoing HP and NP have reported a higher interstage mortality rate of 13–23% for the HP cohort as compared to 2–14% for the NP cohort [32,70,71]. Numerous risk factors in HP, including diastolic run-off away from the systemic and coronary beds, difficulties in adjusting proper pulmonary artery band size and need for reoperation for re-banding, potential restriction of the atrial septal communication requiring reintervention and coronary malperfusion secondary to obstruction to retrograde arch flow, have been associated with increased interstage mortality [32,72,73]. The presence of ventricular dysfunction, restriction of the atrial septum, coronary circulation disorders and stenosis or systemic-pulmonary obstruction in the NP cohort have been associated with interstage mortality [71]. Implementation of parental education and home monitoring programs have improved outcomes in the interstage period for NP cohort patients [74].

Our meta-analysis results revealed a significantly increased mortality rate at 1 year of age for the HP cohort as compared to the NP cohort. A study of HLHS patients from the Pediatric Health Information System has reported increased odds of survival at 1 year for HP as compared to NP [57]. They, however, defined survival in their study as readmission to the hospital at 1 year [57]. The potential advantage of improved survival for the HP cohort in the neonatal period does not seem to be sustained beyond that time. The increased mortality rate at 1 year for the HP cohort may be related to increased interstage mortality [32], an increased need for re-interventions [72], a more tenuous physiologic state [75] and an increased risk of mortality following the comprehensive Stage 2 repair [76,77].

Our meta-analysis revealed a similar risk of mortality at 3 and 5 years in HLHS patients undergoing HP or NP. A risk-adjusted 4-year survival of critical left ventricular outflow tract obstruction patients between 2005 and 2014 in the Congenital Heart Surgeons’ Society revealed significantly better outcomes for patients undergoing NP with a Sano shunt as compared to NP with a BT shunt or HP (76% vs. 60% vs. 61%; *p* < 0.001). The 4-year survival for propensity-matched neonates between HP and NP with a BT shunt, however, was similar (62% vs. 57%; *p* = 0.58) [15]. Likewise, the 5-year survival of HLHS between 2008 and 2012 in the Japan Congenital Cardiovascular Surgery Database reported significantly better outcomes for NP as compared to HP (75.5 vs. 54.0%, log–rank *p* < 0.001) [32]. Studies included in our meta-analysis had the limitation of specifying the type of shunt (Sano shunt or BT shunt) undertaken in the NP cohort patients [36,38,40,41,43,44,45,46,49,51]. The arterial source of pulmonary blood flow in HP and NP with a BT shunt had the potential risk of diastolic runoff leading to compromised coronary perfusion or coronary steal in early life that may be associated with decreased myocardial reserve and hemodynamic compromise [2,78]. A lack of difference in 3-year and 6-year transplant-free survival, however, has been demonstrated in NP undergoing a Sano or BT shunt in the Single Ventricle Reconstruction trial [18,79]

Our meta-analysis revealed high-risk neonates undergoing HP had significantly increased survival rates or need for transplantation as compared to NP following Stage I palliation. Numerous factors, including low birth weight (<2.5 kg), prematurity < 37 weeks of gestation, genetic abnormalities, cardiac conditions such as an intact atrial septum or severe valve insufficiency and noncardiac conditions, have been associated with an increased risk of mortality following Stage 1 palliation in patients undergoing NP [22,80,81]. A delay in the operative procedure or attempts to increase preoperative weight has not demonstrated improved outcomes [82]. Improved outcomes for Stage I HP in these high-risk patients may be related to the avoidance of CPB, cardioplegia and DHCA that mitigate CPB-associated postoperative systemic inflammatory response, varying degrees of ischemic and inflammatory injury to the neonatal myocardium that may or may not be reversible and surgical alterations in cerebral blood flow in the neonatal period when the brain may be more susceptible to neurologic injury [54]. Recent guidelines from the European Association for Cardio-Thoracic Surgery (EACTS) and the Association for European Paediatric and Congenital Cardiology (AEPC) Hypoplastic Left Heart Syndrome Guidelines task force recommend HP for high-risk HLHS patients, including weight < 2.0 kg, the presence of necrotizing enterocolitis, cerebral injury, restrictive foramen ovale, or intact atrial septum [16].

Our meta-analysis revealed a lack of significant differences in risks for interstage mortality or 1-year mortality or need for transplantation among high-risk HLHS patients undergoing HP or NP. The improved survival for the HP cohort in the early period was not sustained in our study. Technical issues of ductal stenting and tightness of pulmonary artery band in HP, frequent re-interventions and an increased risk of mortality following Stage II palliation may be responsible for some of the increased risk of mortality in high-risk patients undergoing HP [29,35,39]. The lack of significant improvement in outcomes in these HLHS patients, however, may be more related to inherent high-risk factors rather than the technical issues related to the surgical procedures [8,15,83]. Analysis of HLHS neonates with birth weight < 2500 g or gestational age < 36 weeks undergoing Norwood and hybrid procedures between 2012 and 2020 revealed birth weight and gestational age to be the most important factors for determining outcomes [8]. Likewise, lower birth weight had a strong association with poor outcomes for the entire cohort as well as for matched-pair comparisons in neonates with critical left ventricular outflow tract obstruction that underwent either NP with Sano shunt, Norwood with a BT shunt or hybrid procedure between 2005 and 2014 at 21 institutions in Congenital Heart Surgeons’ Society (CHSS) [15].

Our meta-analysis revealed an increased risk for morbidity in patients undergoing HP as compared to NP. There was a decrease in the number of HLHS patients undergoing Stage II and Stage III palliation following HP as compared to NP. Factors, including an increased risk of interstage mortality for HP and an increased mortality following comprehensive Stage II repair in HP, may be responsible for this decrease [76,84]. Operative mortality of 11–12.4% has been reported after comprehensive Stage II repair after HP as compared to the mortality of 2.5% for the bidirectional Glen procedure for NP [76,77,84]. In addition, factors such as low birth weight, prematurity, a pre-operative need for mechanical ventilation, a preoperative history of infection or an abnormal neurological condition that are associated with the failure of the successful completion of the Stage III repair may be more prevalent in the HP cohort patients [84].

Our meta-analysis revealed that patients undergoing HP had a significantly increased number of unexpected interventions as compared to NP cohort patients. Pulmonary artery reintervention rates after comprehensive Stage II repair ranged from 46% to 50% at experienced hybrid centers and can be as high as 86% in low-volume centers [5,35,72]. The presence of bilateral pulmonary artery bands in small pulmonary arteries and the duration of the band being more than 90 days are risk factors for multiple interventions [41]. Pulmonary artery interventions include catheter-based balloon dilation with or without pulmonary artery stent placement [35,73,85,86]. In addition, reinterventions for obstruction of the stented arterial duct and repeated percutaneous atrial septostomy for unobstructed interatrial communication are more prevalent in HP cohort patients [47]. Unexpected interventions in NP patients are higher in the Sano shunt cohort as compared to the BT shunt cohort [2,79]. This difference is largely attributable to the higher balloon dilation or stent placement rate in the shunt or a branch of the pulmonary artery and the greater incidence of aorto-pulmonary collateral coiling in the Sano shunt cohort [2,79].

Our meta-analysis revealed a lack of significant difference in the mean pulmonary artery pressures at Stage II and Stage III palliation among patients undergoing HP or NP. The size and the quality of branch pulmonary arteries and their arborization are important for the long-term outcome of the Fontan circulation. All surgical procedures on the pulmonary arteries try to focus on promoting growth and avoidance of distortion and scar tissue. In HP, pulmonary arterial banding induces regional inflammation within the vascular wall and ischemic injury, resulting in the loss of cells in the media that may have important long-term consequences [87]. Technical modifications, including avoidance of tight bands or asymmetrical pulmonary artery bands or longer duration of pulmonary artery bands, may partly overcome issues related to pulmonary arterial growth potential [39,41]. In NP, left pulmonary artery stenosis and central branch pulmonary artery stenosis have been partly overcome by technical modifications [88,89,90]. The Single Ventricle Reconstruction trial did not reveal significant differences in mean left and right branch pulmonary artery pressures at Stage III between the NP Sano shunt and BT shunt [26].

Our metanalysis did not reveal any significant difference in right ventricular dysfunction by echocardiography between HP and NP at Stage II palliation. Preservation of single right ventricular function is a key factor during staged palliation of children with HLHS [18]. Ventricular function in HP patients can be compromised due to the predominantly retrograde perfusion of the ascending aorta and coronary circulation through a small isthmus that limits coronary blood flow reserve [54]. The right ventricle in HP patients, as compared to NP patients, has decreased mechanical efficiency with a higher myocardial oxygen demand [91]. Ventricular function after NP can be affected by myocardial ischemia–reperfusion injury after cardioplegic arrest at Norwood Stage I palliation, ventriculotomy at the site of the construction of RV-PA conduits and volume overloading of the systemic ventricle during the interstage period [2,18]. Serial assessment of qualitative and quantitative right ventricular function by echocardiography can be beneficial to prognosticate outcomes in HLHS patients [92]. In the Single Ventricle Reconstruction trial, the right ventricle ejection fraction was similar in NP between the Sano shunt and BT shunt at Stage II palliation, reduced at Stage III and similar at 12 years of age [2,18,79]. Likewise, quantitative assessment of right ventricular function by echocardiography or by cardiac magnetic resonance at different stages of repair did not reveal significant differences between NP and HP patients [47,53].

Our systemic analysis of studies evaluating neurodevelopmental outcomes in HLHS patients undergoing HP or NP did not reveal significant differences at 1, 2 and 4 years of age. The advantage of HP in the early neonatal period by avoiding neurological risks associated with CPB, DHCA and ACP may be counterbalanced by abnormal cerebral blood flow due to prolonged retrograde perfusion of the aortic arch, the need for more reinterventions and the risk for acquired cerebral abnormalities at a later age of surgery [93,94,95]. Neurodevelopmental outcomes in HLHS patients have been associated with the length of mechanical ventilation, ICU and hospital stays, which tend to be longer for HP patients [65,67]. Neurocognitive deficiency in HLHS may also be related to the presence of pre-existing structural abnormalities in their brain tissue [96,97,98].

Our meta-analysis revealed a significantly longer ICU and hospital stay following Stage I palliation in the neonatal period for HP as compared to NP, although the ICU stay was not significantly different. Previous studies have revealed a variable length of hospital stay following Stage I palliation for the HP and NP cohorts [14,57]. Patients undergoing HP can have prolonged ICU and hospital stays due to an increased number of planned and unplanned interventions, salvage Norwood operation during the same stay, or maintenance on prostaglandin therapy [48]. In addition, HP patients tended to have more high-risk factors that are associated with longer stays [8,99].

Our meta-analysis and scientific review have numerous limitations. First, the analysis is subject to the limitations of publication bias, as in all meta-analyses. The majority of included studies were observational and retrospective, with a small sample size [36,37,38,39,40,41,42,43,44,45,46,47,48,49,50,51,52,53,54,55,56]. They are subject to confounding and additional risks of recall bias, selective outcome reporting and selective analysis reporting biases. Our assessment, however, did not reveal indications of potential publication bias. Second, patient selection and criteria for HP or NP in the neonatal period were subjective in most of the studies [36,37,38,39,40,41,42,43,44,45,46,47,48,49,50,51]. Some of the studies included only high-risk patients for HP. However, there were multiple high-risk factors included in the criteria for comparison. We thus chose to include HLHS patients who underwent HP or NP at the same center/centers that had similar criteria for selection in our analysis to avoid variability in patient selection and type of procedure across different institutions. Third, there was heterogeneity in the HP procedure in different studies. Some underwent ductal stenting versus prostaglandin infusion for maintaining patency of the ductus arteriosus; atrial septostomy was part of the HP in some studies, whereas it was undertaken in the presence of restrictive atrial septum in other studies; the criteria for the tightness of the pulmonary artery band varied and was not well defined in most of the studies, and lastly, some of the centers offered emergent NP or elective NP procedure after 4–6 weeks of age as compared to comprehensive Stage II repair. Fourth, there was a lack of clarity of indication and the number of NP patients that underwent a Sano or BT shunt. Thus, we were unable to compare outcomes in the HP cohort versus NP Sano shunt or BT shunt cohorts, separately. Fifth, there was a lack of studies comparing near-infrared spectroscopy (NIRS) between NP and HP patients. Continuous noninvasive measurement of regional cerebral and somatic NIRS saturations in the early postoperative period can predict outcomes, including early mortality, extracorporeal membrane oxygenation use and cardiac arrest, in HLHS patients [100,101]. Sixth, significant advances and technical innovations have been made in single ventricle palliation surgery, pediatric cardiac anesthesia and intensive care unit management in the past decade that have significantly improved the outcomes in HLHS patients [102,103,104]. It is difficult to delineate their contributory roles in the outcomes of NP and HP patients due to a lack of studies. HLHS patients with preoperative high-risk factors, however, continue to have higher mortality, and standardization of management after NP has not shown improved 1-year outcomes [23,27]. Seventh, the follow-up of HLHS patients after the neonatal period was variable, with missing data over the period, and most of them reported outcomes until 1 year of age. Lastly, one of the major reasons for undertaking HP in the neonatal period is to avoid the risk of exposure to CPB, DHCA, and ACP, which have been associated with poor neurological outcomes. There was a paucity of studies that reported neurodevelopmental outcomes among HP and NP cohorts to see any beneficial value of undertaking HP in the neonatal period. Nevertheless, our metanalysis and scientific review aimed to overcome the small sample sizes, heterogeneity, and limited follow-up in previous studies to offer a more generalizable overview of the available data.

### Future Directions

Prospective, randomized multicenter studies with well-defined selection criteria for HP versus NP and long-term follow-up are required to be undertaken to delineate any beneficial role of HP over NP.

## 5. Conclusions

Outcomes following NP in the neonatal period have reached a plateau in the past decade. HP has been introduced as an alternative procedure to NP to facilitate better outcomes as it limits exposure to CPB, DHCA and altered cerebral perfusion in the fragile neonatal period. Our results, however, reveal an increased risk of mortality and/or transplantation in the interstage period and at 1 year in patients in the HP cohort with a similar risk of mortality and/or transplantation at 3 and 5 years as compared to patients in the NP cohort. In the high-risk neonates, the initial reduction in mortality and/or transplantation in the HP cohort in the neonatal period was not sustained in the interstage period and at 1 year of age. HLHS patients in the HP cohort had more morbidity with a higher number of unexpected interventions, fewer Stage II and Stage III palliation procedures undertaken, longer hospitalization after Stage I and Stage II palliation, and lack of better neurodevelopmental outcomes as compared to patients in the NP cohort.

## Figures and Tables

**Table 1:** Summary of studies included in the meta-analysis.

Study	Publication Year	Study Period	Study Location	HLHS (*n*)	HP (*n*)	NP (*n*)
Lim et al. [36]	2006	2002–2005	Virginia, USA	22	5	17
Pizzaro et al. [37]	2008	2001–2006	Nemours, Wilmington, DE, USA	33	14	19
Sakurai et al. [38]	2009	2004–2007	Fukuoka, Japan	43	18	25
Baba et al. [39] *	2012	2004–2010	Toronto, ON, Canada	110	47	63
Brescia et al. [40]	2013	2007–2012	Saint Louis, MO, USA	40	24	16
Davies et al. [41] ^#^	2014	2001–2013	Nemours, USA	104	50	54
Lloyd et al. [42]	2014	2005–2011	London, UK	138	27	111
Knirsch et al. [43] ^§^	2014	2008–2011	Zurich, Switzerland	26	14	12
DiBardino et al. [44]	2015	2007–2012	San Diego, CA, USA	68	26	42
Dodge-Khatami et al. [45]	2015	2010–2014	Jackson, MS, USA	47	17	30
Nwankwo et al. [46]	2018	2004–2015	Pittsburgh, PA, USA	40	24	16
Latus et al. [47]	2018	2008–2015	Giessen, Germany and London, UK	86	44	42
Sower et al. [48]	2019	2000–2016	Michigan, USA	96	35	61
Erek et al. [49]	2020	2011–2018	Istanbul, Turkey	97	65	32
Ho et al. [50]	2021	2013–2020	Southampton, Leeds, Sheffield, Great Ormond Street, Bristol, UK	186	46	140
Matsunaga et al. [51]	2023	2004–2022	Kitasato, Gunma, Juichi, Japan	46	23	23

* Data collated with Grotenhuis et al. [53], Chetan et al. [54], Honjo et al. [55]; ^#^ Data collated with Davies et al. [52]; ^§^ Data collated with Knisch et al. [56]; HLHS: Hypoplastic Left Heart Syndrome; HP: Hybrid Palliation, NP: Norwood Palliation.

## Data Availability

No new data were created or analyzed in this study. Data sharing is not applicable to this article.

## Supplementary Materials

The following supporting information can be downloaded at: https://www.mdpi.com/article/10.3390/jcm13144244/s1, Figure S1: Funnel plot for studies evaluated for mortality in neonatal period; Figure S2: Funnel plot for studies evaluating interstage mortality: Figure S3: Funnel plot for studies evaluating 1-year mortality.
