# Supplementary material for: Comparison of Morbidity and Mortality Outcomes between Hybrid Palliation and Norwood Palliation Procedures for Hypoplastic Left Heart Syndrome: Meta-Analysis and Systematic Review
Source: J Clin Med. 2024 Jul 20;13(14):4244. doi: 10.3390/jcm13144244 (PMC11277754; doi:10.3390/jcm13144244)
Supplement: Supplementary file 1 [file jcm-13-04244-s001.zip › jcm-3015465-supplementary.pdf]

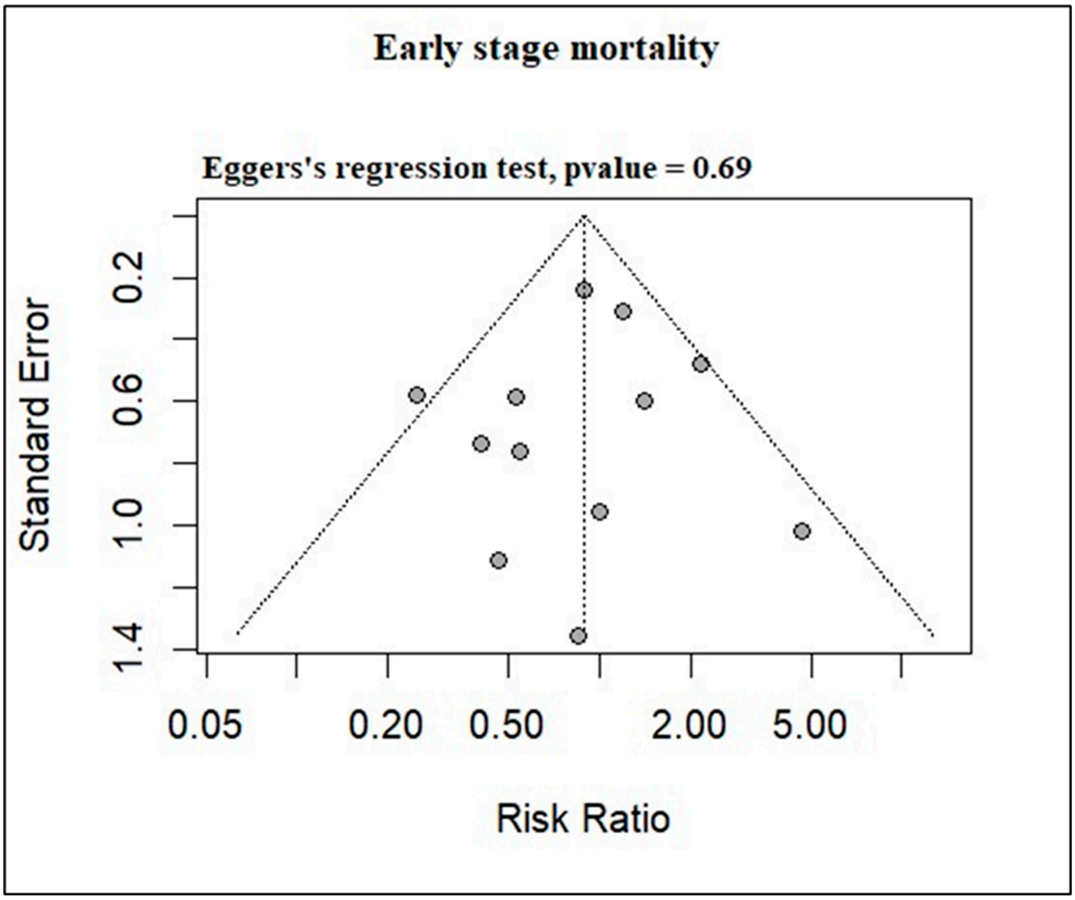

Supplementary Figure S1. Funnel plot for studies evaluated for mortality in neonatal period.

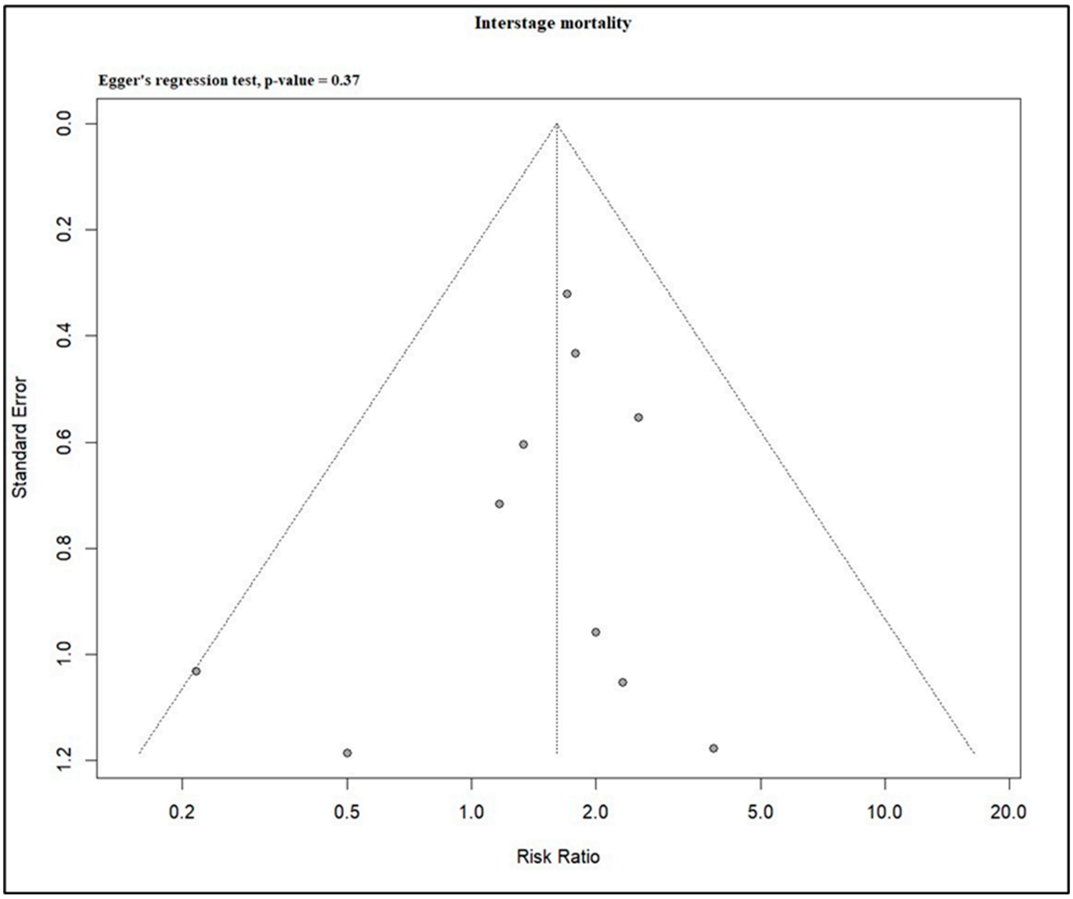

Supplementary Figure S2. Funnel plot for studies evaluating interstage mortality.

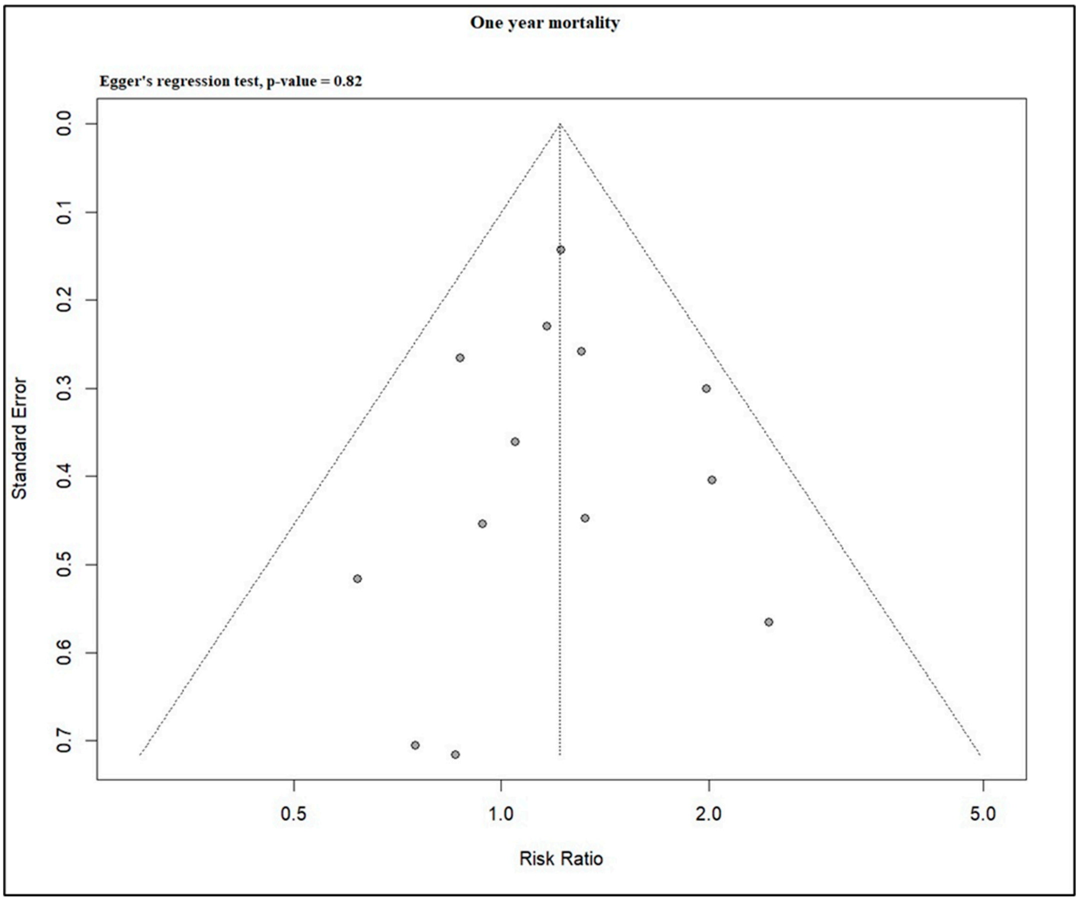

Supplementary Figure S3. Funnel plot for studies evaluating 1-year mortality.
